# Covariance and crossover matrix guided differential evolution for global numerical optimization

**DOI:** 10.1186/s40064-016-2838-5

**Published:** 2016-07-26

**Authors:** YongLi Li, JinFu Feng, JunHua Hu

**Affiliations:** 1 Institute of Aeronautics and Astronautics Engineering, Air Force Engineering University, Room 1, BaLing Road, Baqiao District, Xi’an City, 710038 China; 2 Institute of Equipment Engineering, Armed Police Force Engineering University, Xi’an, 710086 China

**Keywords:** Differential evolution, Numerical and engineering optimization, Crossover matrix, Covariance matrix, Memory population

## Abstract

Differential evolution (DE) is an efficient and robust evolutionary algorithm and has wide application in various science and engineering fields. DE is sensitive to the selection of mutation and crossover strategies and their associated control parameters. However, the structure and implementation of DEs are becoming more complex because of the diverse mutation and crossover strategies that use distinct parameter settings during the different stages of the evolution. A novel strategy is used in this study to improve the crossover and mutation operations. The crossover matrix, instead of a crossover operator and its control parameter CR, is proposed to implement the function of the crossover operation. Meanwhile, Gaussian distribution centers the best individuals found in each generation based on the proposed covariance matrix, which is generated between the best individual and several better individuals. Improved mutation operator based on the crossover matrix is randomly selected to generate the trial population. This operator is used to generate high-quality solutions to improve the capability of exploitation and enhance the preference of exploration. In addition, the memory population is randomly chosen from previous generation and used to control the search direction in the novel mutation strategy. Accordingly, the diversity of the population is improved. Thus, CCDE, which is a novel efficient and simple DE variant, is presented in this paper. CCDE has been tested on 30 benchmarks and 5 real-world optimization problems from the IEEE Congress on Evolutionary Computation (CEC) 2014 and CEC 2011, respectively. Experimental and statistical results demonstrate the effectiveness of CCDE for global numerical and engineering optimization. CCDE can solve the test benchmark functions and engineering problems more successfully than the other DE variants and algorithms from CEC 2014.

## Background

Global optimization has been extensively applied in various science and engineering fields. Unconstrained global optimization is important in optimization. Thus, numerous studies on global optimization have been conducted using various strategies to achieve unconstrained global optimization (Deep et al. 2009; Fan and Yan 2015; Gwizdałła 2012). However, serious challenges in global optimization remain, such as non-linear, non-convex, and non-differential problems.

Differential evolution (DE) is one of the most efficient evolutionary algorithms (EAs) and has wide application in numerous numerical optimization problems in diverse fields (dos Santos Coelho et al. 2014). ED was first introduced by Storn and Price (1995). DE is a population-based optimization algorithm similar to other EAs. This algorithm primarily consists of a mutation operator and a crossover operator (Storn and Price 1997). Each individual in the population in DE is called a target vector. First, a mutant vector is produced by the mutation operator. Then, a trial vector is confirmed by the crossover operator applied to the target and mutant vectors. Finally, the better solution is selected between the trial vector and its target vector according to their objective function values. DE has been successfully demonstrated in various continuous optimization problems in many science and engineering fields because of its simple structure, easy operation, convergence property, quality of solution, and robustness. DE has also been used in robot control (Wang and Li 2011), sensor array interrogation (Venu et al. 2008), cluster analysis (Maulik and Saha 2009), and other applications (Dong et al. 2014; Gundry et al. 2015; Zhang and Duan 2015; Zhang et al. 2015).

DE is sensitive to the choice of the mutation and crossover operators and their two associated control parameters, namely, the crossover control parameter *CR* and scaling factor *F* (Qin et al. 2009). The influence of these factors has been paid much attention, and a series of different DEs has been proposed to improve the optimization performance. Brest et al. (2006) proposed the JDE algorithm, which is a DE with self-adaptive parameter control. In this algorithm, *CR* and *F* are encoded into the chromosome and participate in the evolution. Zhang and Sanderson (2009) improved *F* by Cauchy distribution and *CR* by normal distribution in the parameter-adaptive DE algorithm called JADE. Moreover, self-adaptive equations for *CR* and *F* have been proposed to control their values with increased generation. Qin et al. (2009) proposed another self-adaptive DE called SaDE with a strategy pool as well as different parameter settings. Mallipeddi et al. (2011) proposed the EPSDE algorithm, which is a DE with an ensemble of control parameter and mutation strategies. EPSDE has a distinct trial vector generation strategy pool and controls parameter pool to self-adjust its search strategy along with the iteration process. Wang et al. (2014) introduced the CoBiDE algorithm, which uses a covariance matrix learning strategy based on the current population distribution to initialize the population of DE and a bimodal distribution strategy to control the value of the two control parameters. These DE-based algorithms and other improved DEs have enhanced the optimization performance of DE to some extent. However, the simple structure of standard DE has been considerably changed, resulting in the apparent difficulty in balancing between exploration (searching for better individuals) and exploitation (using the existing material in the population to obtain the best effect) (Fraa et al. 2015).

Thus, we propose a covariance and crossover matrix-guided DE (CCDE) based on several studies (Ghosh et al. 2012; Santucci and Milani 2011; Zhabitsky and Zhabitskaya 2013) to solve these problems. The covariance matrix between the current best individual and several better individuals can reflect the rotation information of the function to some extent. Thus, the covariance matrix is used to guide the generation of new individuals. We introduce the Gaussian distribution that centers the best individuals found in each generation based on the proposed covariance matrix. The crossover operator and its parameter *CR* are simplified and replaced by the crossover matrix, which is a random binary integer-valued matrix composed of 0 and 1. In addition, the memory population *M* is introduced to enhance the exploration of the CCDE and is used to control the search direction of the generation. CCDE has been tested on 30 benchmarks chosen from the IEEE Congress on Evolutionary Computation (CEC) 2014 (Liang et al. 2013) and 5 real-world engineering problems selected from CEC 2011 (Das and Suganthan 2010). The performance of CCDE is compared with those of JADE, SaDE, EPSDE, and CoBiDE, as well as five algorithms from CEC 2014. The experimental and statistical results suggest that the performance of CCDE is better than those of other compared algorithms.

The rest of this paper is organized as follows. Section “DEA” introduces DE briefly. CCDE is presented in section “CCDE”. The experimental results are presented in section “Experimental study”. Finally, section “Conclusion” elaborates the conclusion and future work.

## DEA

DE is a population-based heuristic search algorithm and has four basic processes: initialization, mutation, crossover, and selection.

### Initialization

DE performs an initialization by selecting several points from the search space randomly using Eq. (1), as follows:

1 $$P_{0} = \{ x_{i,0} = (x_{i,1,0} ,x_{i,2,0} ,x_{i,3,0} , \ldots ,x_{i,D,0} ),\quad i = 1,2,3, \ldots ,N\}$$

where *D* denotes the dimension of the population and *N* denotes the population size. The vector element of *x*_*i*,0_ is a random number uniformly distributed in the range [*low*, *up*], where *low* and *up* are the boundaries of the search space.

### Mutation

The standard mutation strategy used in DE is “DE/rand/1” and can be illustrated using Eq. (2), as follows:

2 $$v_{i,G} = x_{r1,G} + F \cdot (x_{r2,G} - x_{r3,G} )$$

where *F* is the scaling factor varied from 0.4 to 1; and *r*_1_, *r*_2_, and *r*_3_ are randomly chosen from [1, *N*]. *i*, *r*_1_, *r*_2_, and *r*_3_ are mutually different. *G* (*G* = 1, 2, 3, …, *Maxgen*) is the current generation. Control parameter *F* is a random value for each individual. A larger *F* is effective for global search, while a smaller *F* is useful for local search.

### Crossover

After mutation, the crossover operator is used by Eq. (3), as follows:

3 $$u_{i,j,G} = \left\{ \begin{array}{ll} v_{i,j,G} , &\quad if\,rand(0,1) \le CR \, or \, j = j_{rand} \\ x_{i,j,G} , &\quad otherwise \end{array} \right.$$

where *CR* is a crossover control parameter or a factor selected from the range [0,1), *i* = 1, 2, …, *N* and *j* = 1, 2, …, *D*. *j*_*rand*_ is an integer value randomly chosen from [1, *N*]. The trial vector *u*_*i,G*_ is generated in the process. *CR* controls the mutation probability. The larger *CR* inherits more elements from the mutant vector.

### Selection

In the selection process, DE chooses the better one between the target vector *x*_*i,G*_ and trial vector *u*_*i,G*_ according to their fitness value using Eq. (4), as follows:

4 $$x_{i,G + 1} = \left\{ {\begin{array}{ll} {v_{i,G} ,} \hfill &\quad {if\;F(v_{i,G} ) \le F(x_{i,G} )} \hfill \\ {x_{i,G} ,} \hfill &\quad {otherwise} \hfill \\ \end{array} } \right.$$

where *F*(*x*) is the fitness value of vector *x*.

## CCDE

CCDE is a novel DE variant designed to be a global minimizer. Unlike the standard DE, CCDE can be explained by dividing its functions into four steps: initialization, selection-I, trial population generation, and selection-II. The trial population is generated by the crossover and covariance matrices. Algorithm 1 shows the general structure of CCDE.
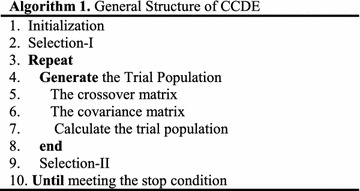


The detailed description of CCDE is presented as follows.

### Initialization

The initialization population *P*_0_ of CCDE is the same as those of other DEs using Eq. (1). Contrary to the other DE variants, *M* in CCDE is used to store the individuals of *P* with rearranged order. Moreover, *M* is used to control the search direction and thus enhance the capability of exploration. Given that *P*_0_ is definite, *M*_0_ is initialized by Eq. (5), as follows:

5 $$M_{0} = \{ y_{i,0} = (y_{i,1,0} ,y_{i,2,0} ,y_{i,3,0} , \ldots ,y_{i,D,0} ),\quad i = 1,2,3, \ldots ,N\}$$

### Selection-I

The fitness values of initialized population *P*_0_ are calculated, and the best individual is stored.

### Generation of trial population

#### Generation of the crossover matrix

This step is the most important process in the CCDE. *M* is adjusted prior to the generation of the trial population to store the previous generation randomly using Eq. (6), as follows:

6 $$M = \left\{ \begin{array}{ll} P, &\quad if\,a < b \\ permuting(M), &\quad otherwise \end{array} \right.$$

where *a* and *b* are random numbers with uniform distribution in the range (0,1). *Permuting* is a function to change the order of individuals in *M* and thus improve its diversity. As a result, the population has a memory capability and is mainly used to improve the performance of exploration.

Then, the crossover matrix (*Cr*) is generated randomly instead of the crossover operator. This matrix is used to determine whether the individuals of *P* must be updated or not. *Cr*_*G*_ is composed of the integer 0 and 1, and initialized by *Cr*_0_ = 0 before the iteration. When *Cr*_*i,j*_ (*i* = 1, 2, 3, …, *N*; *j* = 1, 2, 3, …, *D*) is equal to 0, *x*_*i,j,G*_ remains unchanged. Otherwise, *x*_*i,j,G*_ is updated and generated using Eq. (7), as follows:

7 $$\left\{ \begin{array}{ll} Cr_{{i,u_{{(i:randi\{ D\} )}} } = 1|u = permuting\{ 1,2,3 \ldots ,D\} ,} &\quad if\,rand_{a} < rand_{b} \\ Cr_{i,u} = 1|u = randi\{ D\} , &\quad otherwise \end{array} \right.$$

where *rand*_*a*_ and *rand*_*b*_ are random values selected from the uniform distribution in the range (0,1). *randi*{*D*} is a function to randomly generate the integer value from 1 to *D*. *u*_(*i*:*randi*{*D*})_ represents the vector elements chosen from the vector *u* from the order number *i* to *randi*{*D*}. The elements of *u* are generated by permuting function about the integer numbers {1, 2, 3, … *D*}. In Eq. (7), when *rand*_*a*_ is less than *rand*_*b*_, several vector elements of individual *i* is updated, while the others remain unchanged. Otherwise, only one vector element of individual *i* is changed.

The crossover matrix in this step is mainly used to balance the performance of the exploration and exploitation. The crossover matrix of CCDE is more complex and efficient without *CR* than the crossover operator of other DEs because the diversity of its population is firmly enhanced.

#### Generation of covariance matrix

The best individual found during evolution is used as the leader to guide the search and thus improve the capability of exploitation. The newly generated individual must center the best individuals. The region around the best individual may be considered the potential region to find the next better individual. Therefore, this method is used to generate the covariance matrix. However, considering the avoidance of local optimum and based on the covariance matrix adaptation evolution strategy (CMA-ES) in Hansen and Ostermeier (2001), covariance matrix learning in CoBiDE in Wang et al. (2014), and differential covariance matrix adaptation EA in Ghosh et al. (2012), a novel covariance matrix strategy is proposed by learning from the previous best individual and present population. With the use of this strategy, the covariance matrix inherits the information accumulated during evolution and learns new information from the present population. The covariance matrix is generated by Eq. (8), as follows:

8 $$Co_{G + 1} = rand \cdot Co_{G} + (1 - rand) \cdot \text{cov} (xbest_{1,G} ,xbest_{2,G} , \ldots ,xbest_{\lambda ,G} )$$

where cov(*x*_*best*1,*G*_, *x*_*best*2,*G*_, *x*_*bestλ*,*G*,_) calculates the covariance matrix of the *λ* best individuals in the current generation and $\lambda = \left\lfloor {N/4} \right\rfloor$. The covariance matrix, as indicated by CMA-ES and CoBiDE, is used to guide the generation of trial population and fully utilizes the information of the individuals to improve the convergence speed. However, contrary to CMA-ES and CoBiDE, the information of the *λ* best individuals is considered in the covariance matrix of CCDE.

#### Generation of trial population

The trial population is generated in this step. The covariance matrix is used as a guide to search the region around the best individual by Gaussian distribution and thus improve the exploitation. The exploration is enhanced using the form of “DE/rand/1” with the improved search direction confirmed by memory and target populations. As a result, we choose one of the two strategies randomly to balance the exploration and exploitation, which can be formulated as follows:

9 $$V_{G + 1} = \left\{ {\begin{array}{ll} {P_{G} + Cr_{G} \cdot F \cdot (P_{G} - M_{G} ),} \hfill & {if\;rand_{a} < rand_{b} } \hfill \\ {X_{best,G} + r \cdot {\text{N}}(0,Co_{G} ),} \hfill & {otherwise} \hfill \\ \end{array} } \right.$$

where *M*_*G*_ is a memory population and *P*_*G*_^*’*^ is a random-ordered individual of population *P*_*G*_. *F* is the scale control parameter of DE as illustrated by Eq. (2). *F* = *R* [*R*–C(1, 0.1), where C(1, 0.1) is the Cauchy distribution with local parameter 1 and scale parameter 0.1] (Wang, Lib, and Huang 2014). *X*_*best,G*_ is the current best population consisting of the current best individual. N(0, *Co*_*G*_) is the Gaussian distribution with mean value 0 and variance value *Co*_*G*_. $r = rand_{1} \left( {1 - rand_{2}^{{\left( {1 - \frac{G}{Maxgen}} \right)^{0.7} }} } \right)$ is the adaptive step size, which is similar to that in simulated annealing algorithm (Edmonds 1971). This step size gradually decreases the search range, and *rand* is a random value in [0, 1].

**Fig. 1:**
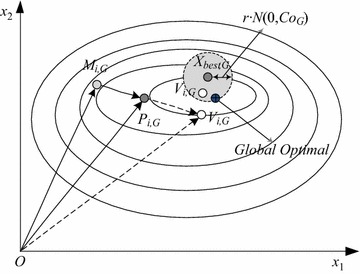
Two dimensional example of an objective function showing its contour lines and the process for generating trial vector in scheme Eq. (9)

From Eq. (9), the search range around the current best individual narrowed with *r* tends to 0 and *G* tends to *Maxgen* to exploit the individual. Meanwhile, falling into local optimum is avoided via the improved mutation operator based on the crossover matrix using a random selection strategy as indicated by Eq. (9). Figure 1 illustrates the generation of the trial vector defined by Eq. (9).

If the *kth* component *v*_*i*,*k*,*G*+1_ of *v*_*i*,k,*G*+1_ is out of the allowed search space, then it is regenerated by Eq. (10), as follows:

10 $$v_{i,k,G + 1} = \left\{ \begin{array}{ll} {up + 0.5 \cdot rand \cdot (v_{i,k,G + 1} - up)}, \hfill &\quad {if\;v_{i,k,G + 1} > up} \\ {low + 0.5 \cdot rand \cdot (low - v_{i,k,G + 1} ),} &\quad {if\;v_{i,k,G + 1} < low} \\ \end{array} \right.$$

where *low* and *up* are the boundaries of the search space.

### Pseudo code for CCDE

The pseudo code can be presented in Algorithm 2 according to the description of CCDE in the previous subsections.
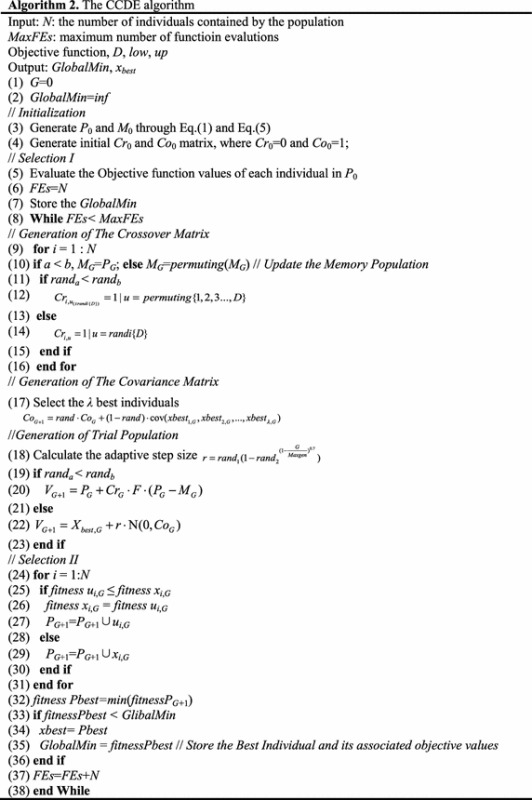


CCDE has a very simple structure as indicated by Algorithm 2. Combining the crossover matrix, covariance matrix, and *M* can achieve a good tradeoff between exploration and exploitation.

## Experimental study

We analyze the performance of our CCDE by conducting a set of experiments as well as a statistical analysis of the experimental results. We use MALLBA 2013a to develop the CCDE algorithm. Non-parametric statistical tests are used in the experimental comparisons because numerical distributions of results sometimes do not follow the conditions of normality and homoscedasticity (García et al. 2009). Therefore, our analyses are mainly focused on the mean errors of 30 or 51 independent runs. Statistical tests are accomplished using the KEEL software, including multi-problem Wilcoxon’s test and Friedman’s test (Alcalá et al. 2009).

We also conduct a series of comparisons with the canonical versions of DE as well as five algorithms from CEC 2014 to clarify the competitiveness of CCDE. All experiments are performed on a computer with 2.9 GHz Intel(R) Core(TM) i5-2310 processor and 4.0 GB of RAM in Windows XP. The set of benchmarks and the parameter settings are described in detail.

### Benchmark functions

A total of 30 benchmark functions developed for IEEE CEC 2014 (Liang et al. 2013) are used, as well as 5 real-world engineering optimization problems selected from IEEE CEC 2011 (Das and Suganthan 2010). The 30 benchmarks are first presented and then the 5 real-world engineering optimization problems are expressed in the following section. The 30 benchmarks can be divided into 4 classes:

1. Unimodal Functions: F1–F3;
2. Multimodal Functions: F4–F16;
3. Hybrid Function: F17–F22; and
4. Composition Functions: F23–F30.

Each function of the above test functions has shift data. F8 and F10 are separable functions, while the rest are non-separable. Some test functions are rotated using different rotation matrices to determine the correlation among variables. The global optima of some test functions are shifted to avoid being at the center of the search space. Contrary to other test functions in previous IEEE CEC, the rotation matrix for each subcomponent is generated from standard normally distributed entries by Gram–Schmidt orthonormalization. The variables in the hybrid functions are randomly divided into subcomponents, and then different basic functions are used for different subcomponents. A local optimum with the smallest bias value is the global optimum in the composition functions, and is set to the origin as a trap for each composition function included in this benchmark suite. Table 1 shows the set of the 30 test functions, which are described in detail in Liang et al. (2013).

**Table 1:** IEEE CEC2014 functions with functions’ features: unimodal (U), multimodal (M), separable (Sep.) and non-separable, rotated (Rot.) and non-rotated, asymmetrical (Asy.) and symmetrical

Function	Name	U/M	Asy.	Sep.	Optimal
F1	Rot. high conditioned elliptic function	U	N	N	100
F2	Rot. bent cigar function	U	N	N	200
F3	Rot. discus function	U	N	N	300
F4	Shif. Rot. Rosenbrock’s function	M	N	N	400
F5	Shif. Rot. Ackley’s function	M	N	N	500
F6	Shif. Rot. Weierstrass function	M	N	N	600
F7	Shif. Rot. Griewank’s function	M	N	N	700
F8	Shif. Rastrigin’s function	M	N	S	800
F9	Shif. Rot. Rastrigin’s function	M	N	N	900
F10	Shif. Schwefel’s function	M	N	S	1000
F11	Shif. Rot. Schwefel’s function	M	N	N	1100
F12	Shif. Rot. Katsuura function	M	N	N	1200
F13	Shif. Rot. HappyCat function	M	N	N	1300
F14	Shif. Rot. HGBat function	M	N	N	1400
F15	Shif. Rot. Exp. Griewank’s + Rosenbrock’s function	M	N	N	1500
F16	Shif. Rot. Exp. Scaffer’s F6 function	M	N	N	1600
F17	Hybrid function 1 (N = 3)	M	N	N	1700
F18	Hybrid function 2 (N = 3)	M	N	N	1800
F19	Hybrid function 3 (N = 4)	M	N	N	1900
F20	Hybrid function 4 (N = 4)	M	N	N	2000
F21	Hybrid function 5 (N = 5)	M	N	N	2100
F22	Hybrid function 6 (N = 5)	M	N	N	2200
F23	Composition function 1 (N = 5)	M	A	N	2300
F24	Composition function 2 (N = 3)	M	N	N	2400
F25	Composition function 3 (N = 3)	M	A	N	2500
F26	Composition function 4 (N = 5)	M	A	N	2600
F27	Composition Function 5 (N = 5)	M	A	N	2700
F28	Composition Function 6 (N = 5)	M	A	N	2800
F29	Composition Function 7 (N = 3)	M	A	N	2900
F30	Composition function 8 (N = 3)	M	A	N	3000
	Search range: [−100, 100]	Dimension: *Dim* = 10 and 30			

Optimal stands for global optimal value

In this section, the mean errors and standard deviations of the function error value [*f*(*x*) − *f*(*x*′)] are calculated over 30 or 51 independent runs for each test function; *x* is the best solution in the population when the algorithm terminates, and *x*′ is the global optimal value. Multi-problem Wilcoxon’s test and Friedman’s test at a 0.05 significance level are performed to test the statistical significance of the experimental results among the compared algorithms. The parameter *N* in this section is set to 100.

### Comparison with other DEs

CCDE is compared with four other DE variants, namely, JADE (Zhang and Sanderson 2009), SaDE (Qin et al. 2009), EPSDE (Mallipeddi et al. 2011), and CoBiDE (Wang et al. 2014). The covariance matrix used in CoBiDE is also based on CMA-ES, and its performance is superior to that of CMA-ES (Wang et al. 2014). Thus, we only choose the CoBiDE, instead of CMA-ES, as the competitor for comparison. The parameter settings for the four algorithms are the same as those in the original papers. JADE adopts self-adaptive parameter setting with *F*_*initial*_ = 0.5 and *CR*_*initial*_ = 0.9. SaDE uses the normal distribution *N* (0.5, 0.3) to produce *F* and the normal distribution *N* (*CR*_*m*_, 0.1) to adjust *CR* self-adaptively. EPSDE sets *F* = 0.9 and *CR* = 0.1. CoBiDE sets *pb* = 0.4 and *ps* = 0.5. In this experiment, *D* of the 30 test functions is set to 10, and each test function independently runs 30 times with 300,000 function evaluations (*FEs*) and error value *Error* = 10^−8^ as the termination criterion.

The experimental results of CCDE and four other algorithms are summarized in Table 2. The portions in italic in Table 2 represent the best results among the algorithms in terms of the optimization of the test functions. CCDE, JADE, SaDE, and CoBiDE exhibit the best performance on the three unimodal functions F1–F3. However, the performance of EPSDE on the three functions is not better than those of the four other algorithms. For the simple multimodal functions F4–F16, CCDE exhibits the best performance on F4–F9 and F11–F14 compared with the four other algorithms. In particular, CCDE can reach the global best value on F4 and F6–F8. CoBiDE shows the best performance on F10 and F15 among all algorithms. EPSDE outperforms the four other algorithms in F16. The outstanding performance of CCDE can be attributed to its proposed strategies that can balance exploration and exploitation. The five algorithms cannot find the global best values for the hybrid functions F17–F22. However, Table 2 shows that the performance of CCDE outperforms the other algorithms on the majority of the test functions, except F18 in which CoBiDE performs better than CCDE. The results of the five algorithms for the composition functions F23–F30, which are the most difficult test functions among the 30 benchmarks, are far from the global optima. Table 2 shows that CCDE is statistically better than the other algorithms on F23–F26 and F28–F30. CoBiDE exhibits the best performance on F27.

**Table 2:** Mean and SD obtained by JADE, SaDE, EPSDE, CoBiDE and CCDE through 30 independent runs on 30 test functions in 10 dimension with 300,000 *FEs*

Function	JADE mean ± SD	SaDE mean ± SD	EPSDE mean ± SD	CoBiDE mean ± SD	CCDE mean ± SD
*Unimodal functions*					
F1	*0.00E+00* *±* *0.00E+00*	*0.00E+00* *±* *0.00E+00*	1.57E+04 ± 3.88E+04	*0.00E+00* *±* *0.00E+00*	*0.00E+00* *±* *0.00E+00*
F2	*0.00E+00* *±* *0.00E+00*	*0.00E+00* *±* *0.00E+00*	2.22E+03 ± 3.24E+03	*0.00E+00* *±* *0.00E+00*	*0.00E+00* *±* *0.00E+00*
F3	*0.00E+00* *±* *0.00E+00*	*0.00E+00* *±* *0.00E+00*	7.65E−04 ± 2.30E−03	*0.00E+00* *±* *0.00E+00*	*0.00E+00* *±* *0.00E+00*
*Multimodal functions*					
F4	1.01E+00 ± 1.87E+00	1.69E+01 ± 1.71E+01	2.64E+01 ± 1.42E+01	3.13E+01 ± 1.06E+01	*0.00E+00* *±* *0.00E+00*
F5	2.01E+02 ± 5.60E−03	2.03E+01 ± 9.91E−02	1.99E+01 ± 9.07E−01	1.95E+01 ± 2.25E+00	*1.91E+01* *±* *3.88E+00*
F6	3.02E−02 ± 1.65E+00	3.76E+01 ± 1.55E+00	9.85E−01 ± 7.78E−01	1.31E−01 ± 3.30E−01	*0.00E+00* *±* *0.00E+00*
F7	3.73E−02 ± 3.83E−02	2.11E−01 ± 1.57E−01	1.47E−01 ± 1.19E−01	3.20E−03 ± 4.00E−03	*0.00E+00* *±* *0.00E+00*
F8	7.01E−01 ± 9.48E−01	1.58E+01 ± 5.93E+00	7.24E+00 ± 3.64E+00	*0.00E+00* *±* *0.00E+00*	*0.00E+00* *±* *0.00E+00*
F9	8.46E+00 ± 3.79E+00	*2.04E+01* *±* *6.50E+00*	7.22E+00 ± 3.79E+00	3.47E+00 ± 1.03E+00	2.49E+00 ± 1.92E+00
F10	6.21E+01 ± 6.82E+01	3.09E+02 ± 2.02E+02	1.67E+02 ± 1.13E+02	*1.52E−01* *±* *7.16E−02*	7.29E+00 ± 1.25E+00
F11	2.93E+02 ± 1.86E+02	6.71E+02 ± 3.37E+02	2.08E+02 ± 1.61E+02	1.91E+02 ± 9.64E+01	*1.73E+02* *±* *1.51E+02*
F12	2.17E−01 ± 1.36E−01	7.19E−01 ± 3.48E−01	2.78E−01 ± 6.29E−02	1.31E−01 ± 3.91E−02	*1.50E−03* *±* *5.90E−03*
F13	1.33E−01 ± 2.81E−02	3.67E−01 ± 1.94E−01	1.14E−01 ± 3.88E−02	5.92E−02 ± 1.60E−02	*8.91E−03* *±* *7.13E−03*
F14	1.26E−01 ± 4.70E−02	3.49E−01 ± 2.02E−01	2.84E−01 ± 1.29E−01	9.18E−02 ± 3.22E−02	*7.91E−02* *±* *2.83E−02*
F15	9.79E−01 ± 3.29E−01	1.48E+00 ± 8.25E−01	7.28E−01 ± 2.76E−01	*6.15E−01* *±* *9.45E−02*	6.69E−01 ± 1.82E−01
F16	2.31E+00 ± 4.25E−01	3.25E+01 ± 2.38E−01	*1.52E+00* *±* *5.31E−01*	2.02E+00 ± 2.67E−01	1.62E+01 ± 6.23E−01
*Hybrid function*					
F17	5.19E+01 ± 6.23E+01	2.96E+02 ± 1.79E+02	2.48E+02 ± 1.76E+02	1.04E+01 ± 5.86E+00	*1.58E+00* *±* *2.57E+00*
F18	1.96E+01 ± 8.62E−01	2.36E+01 ± 1.74E+01	2.51E+01 ± 2.13E+01	*2.37E−01* *±* *2.08E−01*	7.63E−01 ± 8.13E−01
F19	9.48E−01 ± 3.38E−01	2.75E+00 ± 1.73E+00	1.97E+00 ± 1.18E+00	2.61E−01 ± 1.18E−01	*2.32E−01* *±* *3.51E−01*
F20	6.78E−01 ± 5.25E−01	1.66E+01 ± 1.13E+01	1.39E+01 ± 1.29E+01	4.26E−01 ± 1.64E−01	*3.62E−01* *±* *4.69E−01*
F21	1.33E+00 ± 4.16E+00	1.27E+02 ± 1.29E+02	9.97E+01 ± 1.18E+02	4.98E−01 ± 2.25E−01	*4.09E−01* *±* *4.06E−01*
F22	1.07E+01 ± 9.64E+00	2.78E+01 ± 1.42E+01	3.27E+01 ± 3.48E+01	3.18E+00 ± 8.64E−01	*2.72E−01* *±* *2.15E−0*1
*Composition functions*					
F23	*3.29E+02* *±* *0.00E+00*	*3.29E+02* *±* *0.00E+00*	3.29E+02 ± 3.28E−04	*3.29E+02* *±* *0.00E+00*	*3.29E+02* *±* *0.00E+00*
F24	1.20E+02 ± 7.47E+00	1.37E+02 ± 1.06E+01	1.24E+02 ± 2.65E+01	1.09E+02 ± 1.83E+00	*1.08E+02* *±* *2.25E+00*
F25	1.28E+02 ± 1.56E+01	1.86E+02 ± 2.52E+01	1.85E+02 ± 2.79E+02	1.65E+02 ± 4.19E+01	*1.20E+02* *±* *1.79E+01*
F26	*1.00E+02* *±* *3.75E−02*	*1.00E+02* *±* *1.69E−01*	*1.00E+02* *±* *2.79E−02*	*1.00E+02* *±* *1.27E−02*	*1.00E+02* *±* *7.71E−03*
F27	9.39E+01 ± 1.43E+02	4.79E+01 ± 1.16E+02	2.47E+02 ± 1.78E+02	1.15E+02 ± 1.77E+02	*3.82E+01* *±* *1.12E+02*
F28	3.88E+02 ± 5.38E+01	4.57E+02 ± 7.84E+01	4.16E+02 ± 5.57E+01	3.91E+02 ± 3.93E+01	*3.25E+02* *±* *3.38E+01*
F29	2.13E+02 ± 2.63E+01	5.77E+04 ± 3.15E+05	4.25E+05 ± 1.02E+06	2.22E+02 ± 6.66E−01	*1.95E+02* *±* *2.08E+01*
F30	5.06E+02 ± 1.25E+02	8.67E+02 ± 3.98E+02	6.33E+02 ± 1.39E+02	4.66E+02 ± 1.72E+01	*2.40E+02* *±* *4.41E+01*

“Mean” and “SD” indicate the average and standard deviation of the function error values obtained in 30 runs, respectively

We also perform the multi-problem Wilcoxon’s test, which is accomplished using the KEEL software, to check the behavior of the algorithms (Alcalá et al. 2009). Tables 3 and 4 summarize the results of the Wilcoxon’s and Friedman’s tests.  The portions in italic in Tables 3 and 4 represent the best results among the algorithms in terms of the optimization of the test functions. Table 3 shows that CCDE provides higher R+ values than R− values in all cases. Wilcoxon’s test at *α* = 0.05 shows significant differences among CCDE and the competitors. This result indicates that CCDE is significantly better than JADE, SaDE, EPSDE, and CoBiDE on the 30 test functions at *α* = 0.05.

**Table 3:** Results of the multiple-problem Wilcoxon’s test for JADE, SaDE, EPSDE, CoBiDE and CCDE at a 0.05 significance level

Algorithm	R+	R−	*p* value	*α* = 0.05
CCDE vs JADE	*410.0*	25.0	3.368E−06	Yes
CCDE vs SaDE	*430.0*	5.0	3.726E−08	Yes
CCDE vs EPSDE	*448.5*	16.5	3.502E−07	Yes
CCDE vs CoBiDE	*382.5*	82.5	1.399E−03	Yes

**Table 4:** Ranking of JADE, SaDE, EPSDE, CoBiDE and CCDE according to the statistical test of the Friedman test

Algorithms	JADE	SaDE	EPSDE	CoBiDE	CCDE
Uni. Func.	*2.625*	*2.625*	4.5	*2.625*	*2.625*
Multim. Func.	3.3077	4.7692	3.3846	2.1154	*1.4231*
Hyb. Func.	3	4.6667	4.3333	1.8333	*1.1667*
Compos. Func.	2.625	4	4	2.875	*1.5*
Total	2.9833	4.3167	3.9	2.3	*1.5*

Friedman’s test based on the KEEL software is performed to further detect the significant difference among CCDE and the four compared algorithms (Alcalá et al. 2009). Iman–Davenport’s procedure is used as the post hoc procedure. Table 4 summarizes the ranking results of the five algorithms obtained by Friedman’s test. CCDE ranks comparable with JADE, SaDE, and CoBiDE on the unimodal functions and ranks best on the multimodal, hybrid, and composition functions. Thus, CCDE ranks the best on the 30 benchmarks of 10 dimensions compared with JADE, SaDE, EPSDE, and CoBiDE.

**Fig. 2:**
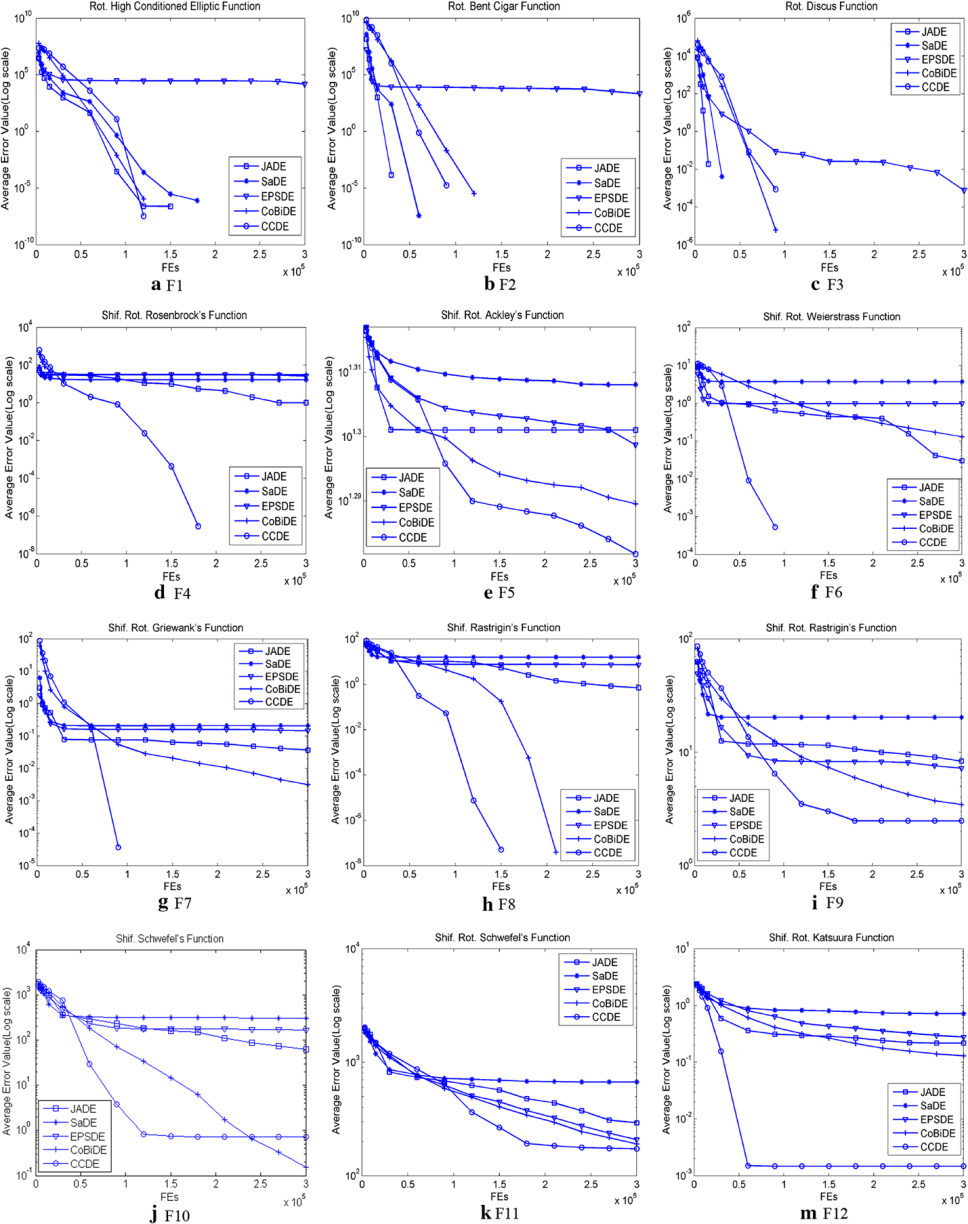
Evolution of the mean function error values derived from JADE, SaDE, EPSDE, CoBiDE and CCDE versus the number of *FEs* from F1 to F12 with *D* = 10

**Fig. 3:**
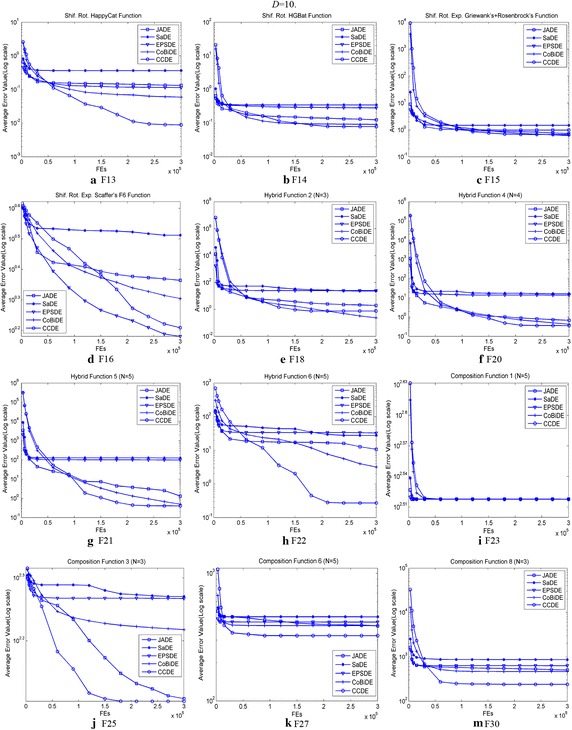
Evolution of the mean function error values derived from JADE, SaDE, EPSDE, CoBiDE and CCDE versus the number of *FEs* on F13-F16, F18, F20-F23, F25, F27 and F30 with D = 10

Figures 2 and 3 illustrate the mean function error values for the 5 algorithms with 30 independent runs for the 24 typical benchmark functions. Figure 2 shows that CCDE can provide better convergence trends for F1, F4–F9, and F11–F12 than the other algorithms. JADE shows the best convergence trends for F2 and F3. CoBiDE presents the best convergence trends for F10. Figure 3 shows that CCDE performs better than the other algorithms on the convergence trends for F13–F15, F20–F22, F25, F27, and F30. EPSDE shows the best convergence preference on F16, whereas CoBiDE performs better on F18. The five algorithms show comparable convergence trends on F23.

### Comparision with CEC 2014 algorithms

CCDE is compared with five algorithms from CEC 2014 in terms of the single-objective real-parameter numerical optimization. These algorithms are all participant algorithms in such special session. They consist of modern real-coded optimizers, hybridizing with local search or using convergence matrix methods. Some of these algorithms follow evolutionary computation or swarm intelligence variants. The five algorithms are convergence matrix learning and search preference algorithm (CMLSP) (Chen et al. 2014); non-uniform mapping in real-coded genetic algorithm (NRGA) (Yashesh et al. 2014); simultaneous optimistic optimization (SOO) (Preux et al. 2014); fireworks algorithm with DE (FWA-DE) (Yu et al. 2014); and OptBees, which is inspired by the collective decision-making of bee colonies (Maia et al. 2014). The experimental results of the compared algorithms are directly taken from (Chen et al. 2014; Yashesh et al. 2014; Preux et al. 2014; Yu et al. 2014; Maia et al. 2014) to ensure fair comparison.

In this experiment, *D* of the 30 test functions is set to 30. Each test function independently runs 51 times with 300,000 *FEs* and error value *Error* = 10^−8^ as the termination criterion for fair comparison. The parameter *N* in CCDE is set to 100.

Table 5 summarizes the experimental results among CCDE and other algorithms in terms of mean errors and standard deviations of 51 independent runs. The portions in italic in Table 5 represent the best results among the algorithms in terms of the optimization of the test functions. CCDE performs better for the majority of the test functions than the five other algorithms.

**Table 5:** Mean and SD obtained by CMLSP, NRGA, SOO, FWA-DE, OptBees and CCDE through 51 independent runs on 30 test functions in 30 dimension with 300,000 *FEs*

Function	CMLSP mean ± SD	NRGA mean ± SD	SOO mean ± SD	FWA-DE mean ± SD	OptBees mean ± SD	CCDE mean ± SD
*A*						
F1	*0.00E+00* *±* *0.00E+00*	5.74E+05 ± 2.89E+05	2.15E+09 ± 0.00E+00	2.76E+05 ± 1.84E+05	8.57E+04 ± 3.04E+05	*0.00E+00* *±* *0.00E+00*
F2	*0.00E+00* *±* *0.00E+00*	9.28E+03 ± 3.95E+03	3.14E+04 ± 0.00E+00	*0.00E+00* *±* *0.00E+00*	*0.00E+00* *±* *0.00E+00*	*0.00E+00* *±* *0.00E+00*
F3	1.23E−08 ± 2.14E−08	4.58E+03 ± 3.81E+03	1.08E+04 ± 5.51E−08	*0.00E+00* *±* *0.00E+00*	8.40E−03 ± 3.77E−02	*0.00E+00* *±* *0.00E+00*
*B*						
F4	1.10E−03 ± 7.60E−03	8.06E+02 ± 3.13E+01	1.09E+02 ± 1.01E−07	2.04E+01 ± 1.93E+01	1.26E+01 ± 1.37E+01	*2.17E−06* *±* *2.61E−06*
F5	1.99E+02 ± 4.93E−05	2.01E+01 ± 1.11E−04	*2.00E+01* *±* *0.00E+00*	2.05E+01 ± 5.41E−02	2.01E+01 ± 1.02E−05	2.02E+01 ± 1.59E−01
F6	3.09E−02 ± 2.21E−01	1.78E+01 ± 2.20E+00	1.89E+00 ± 1.56E−07	1.29E+01 ± 8.33E+00	1.64E+01 ± 3.44E+00	*0.00E+00* *±* *0.00E+00*
F7	0.00E+00 ± 0.00E+00	1.59E−02 ± 1.61E−02	9.96E−01 ± 0.00E+00	8.51E−03 ± 9.92E−03	3.75E−02 ± 3.82E−02	*0.00E+00* *±* *0.00E+00*
F8	9.84E+00 ± 2.96E+01	2.66E+01 ± 7.79E+00	9.25E+01 ± 2.87E−07	1.89E+00 ± 1.57E+00	0.00E+00 ± 0.00E+00	*0.00E+00* *±* *0.00E+00*
F9	8.39E+00 ± 2.39E+00	4.57E+01 ± 1.35E+01	5.97E+01 ± 1.44E−08	5.66E+01 ± 1.09E+01	1.37E+02 ± 3.24E+01	*2.19E+00* *±* *2.37E+00*
F10	1.47E+03 ± 4.78E+02	1.07E+03 ± 4.61E+02	2.31E+03 ± 1.38E−08	*8.53E+00* *±* *2.45E+00*	1.04E+03 ± 2.52E+02	2.24E+01 ± 3.85E+01
F11	1.82E+03 ± 7.56E+02	3.41E+03 ± 6.49E+02	2.15E+03 ± 0.00E+00	2.63E+03 ± 2.51E+02	2.72E+03 ± 5.68E+02	*2.02E+03* *±* *7.91E+02*
F12	*1.46E−04* *±* *4.18E−04*	1.51E+00 ± 7.11E−02	3.01E−02 ± 1.05E−07	3.71E−01 ± 6.73E−02	1.82E−01 ± 6.12E−02	6.29E−04 ± 1.71E−04
F13	*4.81E−02* *±* *2.29E−02*	2.81E−01 ± 5.65E−02	3.50E−01 ± 0.00E+00	3.89E−01 ± 5.57E−02	5.61E−01 ± 1.48E−01	5.54E−02 ± 2.44E−02
F14	3.12E−01 ± 5.13E−02	1.87E−01 ± 2.66E−02	2.91E−01 ± 2.81E−07	2.69E−01 ± 7.83E−02	3.99E−01 ± 2.31E−01	*1.84E−01* *±* *3.51E−02*
F15	*3.02E+00* *±* *1.23E+00*	1.37E+01 ± 4.82E+00	2.25E+01 ± 7.17E−08	7.37E+00 ± 8.55E−01	1.27E+01 ± 6.92E+00	3.25E+00 ± 6.16E−01
F16	1.28E+01 ± 7.05E−01	1.47E+01 ± 6.74E−01	9.86E+00 ± 0.00E+00	1.09E+01 ± 2.74E−01	1.09E+01 ± 6.91E−01	*1.06E+01* *±* *6.34E−01*
*C*						
F17	*9.35E+02* *±* *3.88E+02*	2.35E+05 ± 1.19E+05	2.81E+07 ± 0.00E+00	6.28E+03 ± 6.01E+03	2.74E+04 ± 4.04E+04	1.05E+03 ± 4.01E+02
F18	7.54E+02 ± 1.61E+01	5.51E+02 ± 7.16E+02	2.86E+03 ± 1.38E−07	7.67E+01 ± 3.69E+01	1.96E+02 ± 4.77E+02	*2.17E+01* *±* *1.42E+01*
F19	*3.88E+00* *±* *6.11E−01*	1.37E+01 ± 1.30E+00	1.84E+02 ± 0.00E+00	9.95E+00 ± 1.95E+00	7.89E+00 ± 1.88E+00	4.16E+00 ± 1.08E+00
F20	9.79E+00 ± 4.68E+00	1.14E+04 ± 5.61E+03	3.82E+04 ± 3.67E−08	4.28E+01 ± 2.64E+01	8.53E+02 ± 7.79E+02	*8.59E+00* *±* *3.01E+00*
F21	2.29E+02 ± 1.19E+02	1.81E+05 ± 9.51E+04	1.63E+07 ± 0.00E+00	7.29E+02 ± 9.59E+02	1.74E+04 ± 1.82E+04	*1.72E+02* *±* *1.01E+02*
F22	6.09E+01 ± 5.74E+01	4.07E+02 ± 1.31E+02	1.02E+03 ± 5.74E−07	1.46E+02 ± 8.92E+01	2.32E+02 ± 9.25E+01	*4.68E+01* *±* *2.17E+01*
*D*						
F23	2.00E+02 ± 0.00E+00	3.15E+02 ± 1.41E−03	2.00E+02 ± 0.00E+00	3.14E+02 ± 0.00E+02	3.15E+02 ± 6.67E−02	*2.00E+02* *±* *0.00E+00*
F24	2.00E+02 ± 5.05E−03	2.28E+02 ± 4.25E+00	2.00E+02 ± 0.00E+00	2.26E+02 ± 3.63E+00	2.36E+02 ± 5.47E+00	*2.00E+02* *±* *9.31E−04*
F25	2.00E+02 ± 0.00E+00	2.11E+02 ± 1.70E+00	2.00E+02 ± 0.00E+00	2.00E+02 ± 1.99E−01	2.01E+02 ± 1.69E−01	*2.00E+02* *±* *1.31E−03*
F26	1.24E+02 ± 4.27E+01	1.00E+02 ± 9.33E−02	2.00E+02 ± 0.00E+00	1.00E+02 ± 5.41E−02	1.01E+02 ± 1.72E−01	*1.00E+02* *±* *2.54E−02*
F27	2.00E+02 ± 0.00E+00	5.88E+02 ± 1.72E+02	2.00E+02 ± 0.00E+00	4.01E+02 ± 3.09E+01	4.02E+02 ± 9.77E−01	*2.00E+02* *±* *0.00E+00*
F28	2.00E+02 ± 0.00E+00	1.59E+03 ± 5.76E+02	*2.00E+02* *±* *0.00E+00*	3.93E+02 ± 1.47E+01	4.31E+02 ± 1.52E+01	3.42E+02 ± 1.21E+01
F29	*2.00E+02* *±* *0.00E+00*	1.32E+03 ± 2.09E+02	*2.00E+02* *±* *0.00E+00*	2.11E+02 ± 2.93E+00	2.16E+02 ± 1.18E+00	2.06E+02 ± 1.17E+00
F30	2.00E+02 ± 0.00E+00	2.89E+03 ± 5.49E+02	2.00E+02 ± 0.00E+00	4.53E+02 ± 1.98E+02	5.93E+02 ± 9.87E+01	*2.00E+02* *±* *0.00E+00*

“Mean” and “SD” indicate the average and standard deviation of the function error values obtained in 30 runs, respectively

Wilcoxon’s and Friedman’s tests are performed to further detect significant differences among CCDE and the five competitors (Alcalá et al. 2009). Tables 6 and 7 summarize the results of these tests. The portions in italic in Tables 6 and 7 represent the best results among the algorithms in terms of the optimization of the test functions.

**Table 6:** Results of the multiple-problem Wilcoxon’s test for CMLSP, NRGA, SOO, FWA-DE, OptBees and CCDE at a 0.05 significance level

Algorithm	R+	R−	*p* value	*α* = 0.05
CCDE vs CMLSP	296.0	169.0	1.9808E−01	No
CCDE vs NRGA	*432.0*	3.0	1.8626E−08	Yes
CCDE vs SOO	*383.0*	52.0	1.3914E−04	Yes
CCDE vs FWA-DE	*444.0*	21.0	8.326E−07	Yes
CCDE vs OptBees	*458.5*	6.5	3.0739E−08	Yes

**Table 7:** Ranking of CMLSP, NRGA, SOO, FWA-DE, OptBees and CCDE according to the statistical test of the Friedman test at a 0.05 significance level

Algorithms	CMLSP	NRGA	SOO	FWA-DE	OptBees	CCDE
Uni. Func.	*2.375*	4.625	5.375	3	3.25	*2.375*
Multim. Func.	2.8077	4.5	4.0769	3.6538	4.1923	*1.7692*
Hyb. Func.	2.1667	4.8333	6	3	3.6667	*1.3333*
Compos. Func.	*2.3125*	5.3125	2.4375	3.5625	5.0625	*2.3125*
Total	2.4667	4.8333	4.2167	3.4167	4.2167	*1.85*

The R+ values in Table 6 show that CCDE has better statistical performance than CMLSP, NRGA, SOO, FWA-DE, and OptBees. Wilcoxon’s test at *α* = 0.05 show significant differences among CCDE and the competitors, except for CMLSP. Table 7 shows that CCDE and CMLSP rank the best for the unimodal functions with 30 dimension variables. CCDE ranks the best for the multimodal, hybrid, and composition functions. Thus, CCDE ranks first on the 30 test functions.

**Fig. 4:**
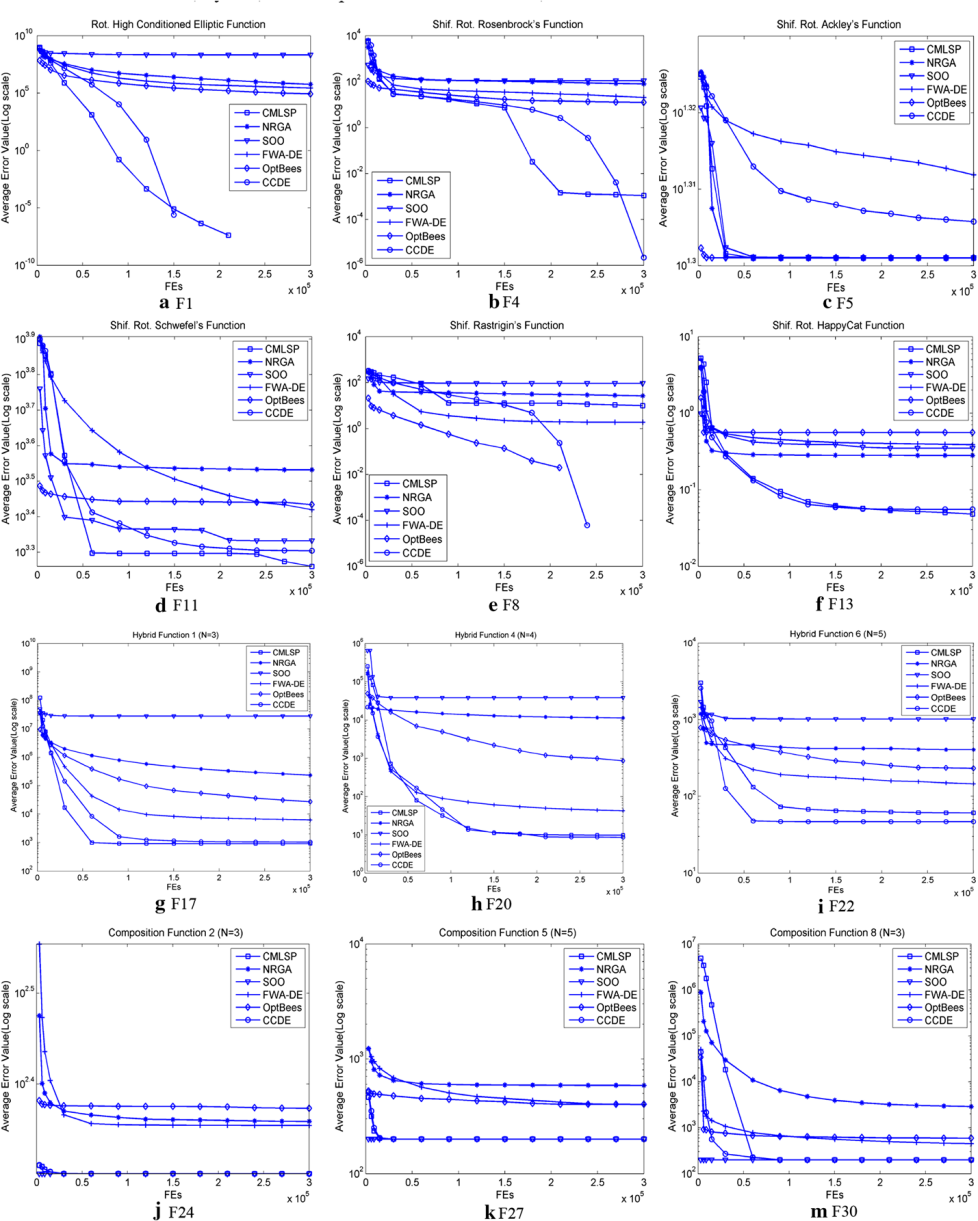
Evolution of the mean function error values derived from CMLSP, NRGA, SOO, FWA-DE, OptBees and CCDE versus the number of *FEs* on 12 test functions with *D* = 30 selected from IEEE CEC2014

Figure 4 illustrates the trace progress for typical test functions with 30 dimension variables.

### Real-world application problems

In addition to the 30 benchmarks in the previous sections, 5 real-world engineering optimization problems from IEEE CEC2011 are selected to evaluate the performance of CCDE in this subsection. These five real-world engineering optimization problems (denoted as RP_1_–RP_5_) are the parameter estimation for frequency-modulated sound waves (T01 in CEC 2011), Tersoff Potential Function Minimization (T06), Spread Spectrum Radar Polly Phase Code Design (T07), Circular Antenna Array Design Problem (T10), Static Economic Load Dispatch Problem (T11.4), and Spacecraft Trajectory Optimization Problem (T13) (Das and Suganthan 2010). These problems have diverse complex characteristics and are very difficult to solve. Detailed descriptions of the five problems can be found in (Das and Suganthan 2010). The parameters of CCDE and other compared DEs are the same with those for the 30 benchmarks. A total of 30 independent runs are performed for each problem, with 150,000 *FE*s as the termination criterion.

Table 8 summarizes the means and standard deviations of the objective function values over 30 independent runs for each problem. Wilcoxon’s and Friedman’s tests at a 0.05 significance level are implemented on the experimental results using KEEL software to draw statistically sound conclusions (Alcalá et al. 2009). Table 9 shows that CCDE has higher R+ values than the other algorithms in all problems. Moreover, *p* values are less than 0.5 in all cases, except for CCDE versus CoBiDE. In addition, CCDE has the best ranking according to Table 10. The portions in italic in Tables 8, 9 and 10 represent the best results among the algorithms in terms of the optimization of the test functions.

**Table 8:** Mean and SD obtained by JADE, SaDE, EPSDE, CoBiDE and CCDE through 30 independent runs on 5 engineering optimization problems with 150,000 FES with 150,000 *FEs*

Problem	JADE mean ± SD	SaDE mean ± SD	EPSDE mean ± SD	CoBiDE mean ± SD	CCDE mean ± SD
RP_1_	9.62E−02 ± 4.08E−02	*0.00E+00* *±* *0.00E+00*	5.06E−02 ± 3.17E−02	*0.00E+00* *±* *0.00E+00*	*0.00E+00* *±* *0.00E+00*
RP_2_	6.52E−01 ± 1.63E−01	1.69E+00 ± 3.31E−01	3.35E+00 ± 7.27E+00	*6.11E−01* *±* *9.71E−01*	6.27E−01 ± 1.01E−01
RP_3_	7.98E−01 ± 9.12E−01	7.66E−01 ± 1.94E−01	7.58E−01 ± 5.58E−01	7.02E−01 ± 4.37E−01	*6.93E−01* *±* *1.59E−01*
RP_4_	−2.13E+01 ± 1.45E+00	−2.15E+01 ± 2.42E−01	−2.14E+01 ± 1.02E−01	−2.15E+01 ± 1.97E−01	*−2.16E+01* *±* *1.76E−01*
RP_5_	1.54E+01 ± 2.12E+00	1.56E+01 ± 1.85E+00	1.43E+01 ± 2.16E+00	1.42E+01 ± 1.75E+00	*1.41E+01* *±* *1.21E+00*

“Mean” and “SD” indicate the average and standard deviation of the function error values obtained in 30 runs, respectively

**Table 9:** Results of the multiple-problem Wilcoxon’s test for JADE, SaDE, EPSDE, CoBiDE and CCDE at a 0.05 significance level

Algorithm	R+	R−	*p* value	*α* = 0.05
CCDE vs JADE	*15.0*	0.0	3.0971E−02	Yes
CCDE vs SaDE	*10.0*	0.0	4.4610E−02	Yes
CCDE vs EPSDE	*15.0*	0.0	3.0971E−02	Yes
CCDE vs CoBiDE	8.0	2.0	2.0124E−02	No

**Table 10:** Ranking of JADE, SaDE, EPSDE, CoBiDE and CCDE according to the statistical test of the Friedman test at a 0.05 significance level

Algorithms	Ranking
JADE	4.4
SaDE	3.5
EPSDE	3.8
CoBiDE	1.9
CCDE	*1.4*

Therefore, these experimental results verify the potential of CCDE in real-world applications.

## Conclusions

The number of works in evolutionary computation involving the solution of difficult optimization problems has been increasing in recent years. DE is an efficient and robust EA and is a hotspot in this field. CCDE, a DE variant based on strategies guided by the crossover and covariance matrices, is proposed in this paper to improve the performance of DE and simplify its structure.

In CCDE, the classical crossover operation and its associated *CR* in DE is simplified by the crossover matrix, which is a binary integer-valued (0, 1) matrix of size *N* × *D* computed by the random generation equation. Improvement is performed to enhance the exploration capability by increasing the diversity of the population. The covariance matrix generated by the *λ* best individuals is used to fully utilize the information for the best individuals and randomly search the region around the best individual by Gaussian distribution. Accordingly, the exploitation capability is improved. In addition, *M* is introduced to store the previous generation and control the search direction. As a result, the diversity of the population is enhanced. CCDE has been tested on 30 benchmark test functions developed for IEEE CEC 2014 and 5 complex real-world engineering optimization problems selected from IEEE CEC 2011. The experimental and statistical results suggest that the performance of CCDE is better than those of the four other DE variants and five algorithms from CEC 2014. CCDE shows high-quality solution and robustness for the tested benchmark functions and real-world engineering problems.

Future studies can extend CCDE by applying the algorithm to various classes of problems, such as multi-objective optimization and constrained optimization problems. The method of CCDE and overall comparison with other evolution algorithms can also be comprehensively studied.
